# Elevated insulin signaling disrupts the growth and differentiation pattern of the mouse lens

**Published:** 2007-03-26

**Authors:** Leike Xie, Huiyi Chen, Paul A. Overbeek, Lixing W. Reneker

**Affiliations:** 1 Department of Ophthalmology, University of Missouri School of Medicine, Columbia, MO; 2 Department of Molecular and Cell Biology, Baylor College of Medicine, Houston, TX

## Abstract

**Purpose:**

Insulin and insulin-like growth factors (IGFs) are putative regulators of cell proliferation and differentiation during lens development. Transgenic mice that overexpress IGF-1 in the lens have been previously described. To further understand the ocular functions of this growth factor family, the in vivo effects of insulin expression on lens development were investigated using transgenic mice.

**Methods:**

Expression of insulin receptor (IR) and IGF-1 receptor (IGF-1R) in mouse lens was examined by reverse-transcriptase-polymerase chain reaction (RT-PCR) and in situ hybridization. Transgenic mice that overexpress insulin in the lens were generated using two different promoters: a fiber-cell specific αA-crystallin (αA) promoter and a modified αA-promoter linked to the chicken δ1-crystallin enhancer (called the δenαA promoter). The δenαA promoter is active in both lens epithelial and fiber cells. The lens phenotypes were analyzed by histology and immunohistochemistry. Protein expression was examined by western blotting.

**Results:**

Normal mouse lenses express both the insulin receptor (IR) and the IGF-1 receptor (IGF-1R), and their expression is highest at the lens periphery where the germinative and transitional zones are located. In transgenic mice, insulin expression in the lens induced cataract formation. The severity of the cataracts reflected the level of transgene expression, independent of the type of promoter used. In severely affected families, the spherical shape of the lens was altered and the lenses were smaller than normal. Histological analysis showed no evidence of premature differentiation of the anterior epithelial cells. In contrast to the IGF-1 mice, insulin transgenic mice exhibited an anterior shift in the location of the germinative and transitional zones, leading to a reduction of the lens epithelial compartment. Additional alterations included expansion of the lens transitional zone, variable nuclear positioning in the lens bow region, and inhibition of fiber cell denucleation and terminal differentiation.

**Conclusions:**

Elevated intraocular insulin does not enhance proliferation nor induce differentiation of mouse lens epithelial cells. Since an increase in IGF-1 causes a posterior shift of the lens geminative and transitional zones, while an increase in insulin causes an anterior shift of these zones, our results suggest that these two growth factors may work together to control the location of this structural domain during normal lens development. Our data also suggest that increased insulin-signaling activity in the lens can antagonize the endogenous signals that are responsible for fiber cell maturation and terminal differentiation.

## Introduction

The lens is a highly organized and polarized tissue. Growth and development of the lens depend on proper spatial regulation of cell proliferation and differentiation [1-3]. The central lens epithelial cells are relatively quiescent while the epithelial cells near the periphery (the germinative zone) show a higher proliferative index. As the epithelial cells in the germinative zone proliferate, cells posterior to this zone are pushed into the transitional zone where they are induced to elongate and differentiate into the lens fiber cells. The newly differentiated fiber cells accumulate in a concentric manner on top of the previously differentiated fiber cells in the bow region. As the immature fiber cells migrate toward the lens core, their nuclei and intracellular organelles degrade to result in mature fiber cells [4].

Growth factors have been implicated as regulators of cell proliferation and differentiation during lens development [1,3]. Gain-of-function studies in mice show that fibroblast growth factors (FGFs) can function as differentiation inducers, while platelet-derived growth factors (PDGFs) function mainly as mitogens [5-8]. The function of insulin and insulin-like growth factors (IGFs) is unclear. Experiments in chicken lens epithelial explants show that insulin and IGFs can induce epithelial-to-fiber differentiation, as measured by cell elongation and an increase in δ-crystallin synthesis [9,10]. In contrast, insulin and IGFs were weak differentiation factors for rat lens epithelial cells in explant cultures. However, these growth factors appeared capable of enhancing or maintaining the differentiation phenotype induced by FGF [11-13]. In transgenic mice generated to overexpress IGF-1 in the lens by means of the mouse αA-crystallin promoter [14], lens epithelial cells were found to not undergo premature differentiation, which is consistent with the observation that IGF-1 itself is not a differentiation inducer in rodent lens explants. Interestingly, the lens growth pattern in IGF-1 mice was perturbed and the lens epithelial compartment was extended more posteriorly than in normal mice.

Insulin and IGF-1 act through two related cell surface receptors, insulin receptor (IR) and IGF-1 receptor (IGF-1R) [15]. These two receptors have a high degree of sequence similarity and both are expressed in most mammalian cell types, although at different levels [16]. Each receptor has a high affinity for its cognate ligand, whereas the binding affinities of insulin to IGF-1R and IGF-1 to IR are about 100 fold lower [17,18]. Interestingly, although IR and IGF-1R are highly conserved from a structural standpoint and share common intracellular signaling pathways, their in vivo physiological functions are quite distinct. IR is primarily important for fuel metabolism, while IGF-1R is primarily important for cell growth [19,20]. There are two main intracellular pathways that are activated by insulin and IGF-1 receptors: the insulin receptor substrate (IRS)-phosphatidylinositol 3-kinase (PI3K) pathway and the Ras-mitogen-activated protein kinase (MAPK) pathway [20-22]. The IRS-PI3K pathway leads to the activation of the three Akt isoforms [23]. Activated Akt can phosphorylate glycogen synthase kinase 3 (GSK3) [24], pro-apoptotic protein BAD [25,26], and transcription factor FOXO [27-29], leading to stimulation of glycogen synthesis, cell survival, and gene expression, respectively. Activation of the Ras-MAPK pathway occurs through the recruitment of the SH2-domain proteins Shc, Grb2, and the exchange factor SOS (son of sevenless) [30,31]. The current consensus, which probably constitutes an oversimplified model, is that the acute metabolic effects of insulin require activation of the IRS-PI3K pathway, while stimulation of cell growth and proliferation requires the Ras-MAPK cascade.

Gene knockout experiments have provided further evidence that IR and IGF-1R have overlapping yet diverse functions in embryonic development [15,32]. Mice lacking either IR or IGF-1R are born with growth retardation of different severity. IGF-1R-null newborn mice are 45% of normal weight, whereas IR-null mice are 90% of normal weight [33-37]. IGF-1R null mice are born with multiple abnormalities, including hypoplastic muscles, delayed bone development, and thin epidermis, and die immediately after birth [33,34]. In contrast, IR null mice develop severe diabetes at an early postnatal age and die of diabetic ketoacidosis, suggesting that IR action is essential for postnatal fuel metabolism and this function cannot be substituted by IGF-1R [35]. Lens defects have not been reported in these gene knockout mice.

Lens epithelial cells in explants respond to either IGF-1 or insulin in a similar fashion [12]. To gain further understanding of the function of these growth factors in lens development, we generated transgenic mice that overexpress insulin in the lens with two different promoters: the mouse αA-crystallin promoter and the newly developed δenαA-promoter. With the αA-crystallin promoter, transgene expression was restricted to the lens fiber cells, whereas the δenαA-promoter directed expression in both lens epithelial and fiber cells [38]. Analysis of the transgenic mice has revealed that the severity of the lens defects was dependent on the transgene expression level rather than the cell-specific expression pattern. Like IGF-1, insulin expression did not induce premature differentiation of lens epithelial cells [14]. Interestingly, insulin expression caused an anterior shift and expansion of the transitional zone and an inhibition of fiber cell terminal differentiation.

## Methods

### RNA isolation and RT-PCR

All animals were used in accordance with the Association of Research in Vision and Ophthalmology (ARVO) Statement for the Use of Animals in Ophthalmic and Vision Research, which is comparable to the animal care guidelines of the Institute of Laboratory Animal Research. Wild type (FVB/N) mouse lenses were obtained at embryonic day 15.5 (E15.5) or at birth (P0), and homogenized in TRI REAGENT (Sigma, St. Louis, MO) to extract total RNA, following the manufacturer's instructions. Reverse transcriptase-polymerase chain reaction (RT-PCR) was performed on 10 ng total lens RNA using the OneStep RT-PCR kit from Qiagen (Valencia, CA). For mouse IGF-1R (GenBank NM_010513), sense (5'-GAG GAC TGT CAT CTC CAA CC-3') and antisense (5'-CGA TTC TTT CAC GCA TAC TTG C-3') primers were used to amplify a 778 base pair (bp) cDNA fragment. For mouse IR (GenBank NM_010568), the sense (5'-GAA GAT CAC CCT TCT TCG AG-3') and antisense (5'-CAG TGA GTC TCT CTG GAC AG-3') primers generated a fragment of 736 bp. PCR products were run on a 2% agarose gel and stained with ethidium bromide for visualization under ultraviolet (UV) light. The PCR fragments were cloned into the pCRII-TOPO cloning vectors (Invitrogen, Carlsbad, CA) and sequenced to verify their identities. These plasmids were also used to generate riboprobes for in situ hybridization to detect the expression patterns of IR and IGF-1R in the mouse lens.

### Histology

Embryonic heads and postnatal eyes were removed from mice and fixed overnight in 10% neutral buffered formalin. The samples were briefly rinsed twice with phosphate buffered saline (PBS), dehydrated in a series of increasing concentrations of ethanol, cleared in xylene, and embedded in paraffin. Sections were cut at 5 μm, de-waxed with xylene, rehydrated with a series of decreasing concentrations of ethanol, and processed for hematoxylin and eosin (H&E) staining, in situ hybridization, or immunohistochemistry.

### In situ hybridization

The procedure for in situ hybridization was described previously [39]. Briefly, radioactive ^35^S-labeled riboprobes were synthesized in vitro from linearized pCRII-TOPO plasmids containing the cDNA for either IR or IGF-1R. After overnight hybridization, the slides were washed, digested with RNase A, and washed again to remove unhybridized probes. Slides were coated with Kodak NTB-2 emulsion and exposed at 4 °C for a week. After developing the slides in the Kodak D-19 developer, the sections were counterstained with hematoxylin. The bright- and dark-field images were captured by a CCD camera.

### BrdU incorporation assay

Pregnant females were injected intraperitoneally with 100 ng of 5-bromo-2'-deoxyuridine (BrdU; Sigma, St. Louis, MO) and 6.7 ng of fluoro-deoxyuridine (FldU, Sigma) per gram of body weight. After 1 h, mice were sacrificed and embryos were collected for BrdU immunohistochemistry, as described previously [40].

### Immunohistochemistry

Immunostaining was performed as previously described [40,41]. Briefly, tissue sections were incubated with blocking solution (5% non-immune serum in phosphate buffered saline [PBS]) for 30 min at room temperature and then with primary antibodies overnight at 4 °C. The source and dilution ratio for each primary antibody are as follows: anti-BrdU (1:100; Dako, Carpinteria, CA), anti α-, β- or γ-crystallins (gifts from Dr. Samuel Zigler at the National Eye Institute; 1:500, 1:1000, or 1:2000, respectively), anti-cytochrome c oxidase (COX) subunit I (catalog number A6403, 1:200; Molecular Probes, Eugene, OR), anti-protein disulfide isomerase (PDI, 1:200; Stressgen, Victoria, BC Canada), anti-phospho-ERK (pERK) and anti-Shc (1:100; Cell Signaling, Danvers, MA). After washing with PBS, sections were incubated with biotinylated (Vector Laboratories) or fluorescein-conjugated (Molecular Probes) secondary antibodies for 1 h at room temperature and then washed with PBS twice. For immunofluorescent staining, cell nuclei were stained with 300 nM 4',6-diaminino-2-phenylindole (DAPI; Molecular Probes) for 10 min at room temperature. Sections were rinsed twice with PBS, mounted in coverslips with 50% glycerol in PBS, and examined under UV illumination. Alternatively, for color detection of immunogens, an avidin-biotin-peroxidase complex (ABC) method was used (kit PK-4002; Vector Laboratories, Burlingame, CA) with diaminobenzidine-H_2_O_2_ as a substrate (Sigma). Sections were counterstained with hematoxylin.

### Western blot analysis

Newborn mouse lenses were isolated and homogenized in RIPA buffer containing 1% NP-40, 0.5% sodium deoxycholate, 0.1% SDS, 0.02% sodium orthovanadate, and 2 mM DTT in PBS. Protease inhibitors (Set III kit from Calbiochem, San Diego, CA) and phosphatase inhibitors (Set II kit from Calbiochem) were added freshly each time. After centrifugation at 10,000 rpm for 5 min, the supernatant was removed and an aliquot was taken for protein determination using the BCA method [42]. For crystallin assays, 10 μg of protein were denatured in 2X SDS sample buffer and boiled for 5 min before loading. For other antigens reported in this study, 100 μg of total lens protein were used. Proteins were separated on 4-12% gradient gels by SDS-PAGE and transferred onto a polyvinylidene difluoride (PVDF) membrane (Invitrogen). The blots were immersed in blocking buffer for 1 h, and then incubated with the primary antibody at 4 °C overnight. The dilution ratios for anti α-, β-, and γ-crystallin primary antibody were 1:3000, 1:6000, and 1:10,000, respectively. Antibodies against ERK, pERK, Akt, pAKT (Cell Signaling), and PDI (Stressgen) were diluted according to the manufacturer's instructions. After washing, the blots were incubated with secondary antibody for 1 h at room temperature, and the antibodies were detected by enhanced chemiluminescence (ECL). Western blotting against β-actin (Sigma) was used as a control to assess equal protein loading.

## Results

### IR and IGF-1R expression in mouse lens

**Figure 1:**
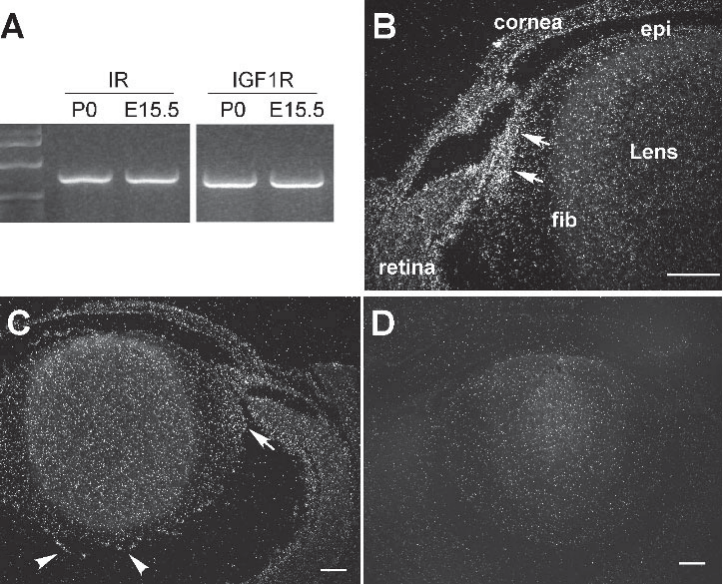
Expression of IR and IGF-1R in mouse lens. **A**: RT-PCR was used to detect IR and IGF-1R expression in embryonic day 15.5 (E15.5) and newborn (P0) mouse lenses. Expression patterns of IGF-1R (**B**) and IR (**C**) in newborn mouse lens were detected by in situ hybridization. IR sense probe was used as a negative control to monitor the background signal (**D**). Both IGF-1R and IR are expressed in the peripheral region of the lens (arrows in **B** and **C**). The IGF-1R signal was stronger than IR in the lens. Other tissues in the eye, including the cornea and retina, also express IGF-1R and IR. A high level of IR mRNA was found in the blood vessel cells surrounding the lens (arrowheads in **C**). Abbreviations used: epi, lens epithelial cells; fib, lens fiber cells. Scale bars in all the figures represent 100 μm.

Lens epithelial cells in explant cultures respond to both insulin and IGF stimulation, implying that these cells express receptors and signaling components for these growth factors [12,13,43]. IR and IGF-1R transcripts were detected by RT-PCR in E15.5 and newborn lenses (Figure 1A). To examine the expression patterns of these two receptors within the lens, in situ hybridizations were done using newborn mouse lenses (Figure 1B-D). IR and IGF-1R were expressed mainly in the lens germinative and transitional zones. Signals for IR were weaker than those for IGF-1R (Figure 1B,C). IR and IGF-1R expression was also detected in the developing retina, iris, ciliary body, and cornea (Figure 1B,C). Interestingly, the vascular endothelial cells outside the lens expressed IR at a higher level than IGF-1R (arrowheads in Figure 1C).

### Lens defects in insulin-expressing transgenic mice

The two types of promoters used in this study were the mouse αA-crystallin promoter (αA) and a modified αA-promoter linked to the δ1-crystallin enhancer (δenαA). In the two αA-insulin transgenic families (OVE441 and OVE442), transgene mRNA was detected only in the lens fiber cells [38]. In contrast, transgenic mice made with the modified δenαA promoter showed high levels of transgene expression in both lens epithelial and fiber cells [38]. A total of six δenαA-insulin transgenic families were established and named LR12, LR13, LR14, LR16, LR20, and LR22.

Transgenic mice from each family except LR20 had visible cataracts when they opened their eyes at postnatal day 14 (P14). Mice from family LR20 eventually developed cataracts at 4 months of age. In situ hybridization showed that the transgene was weakly expressed in this family (data not shown). In the families with the highest levels of transgene expression, OVE442 and LR22, the eyes were microphthalmic.

**Figure 2:**
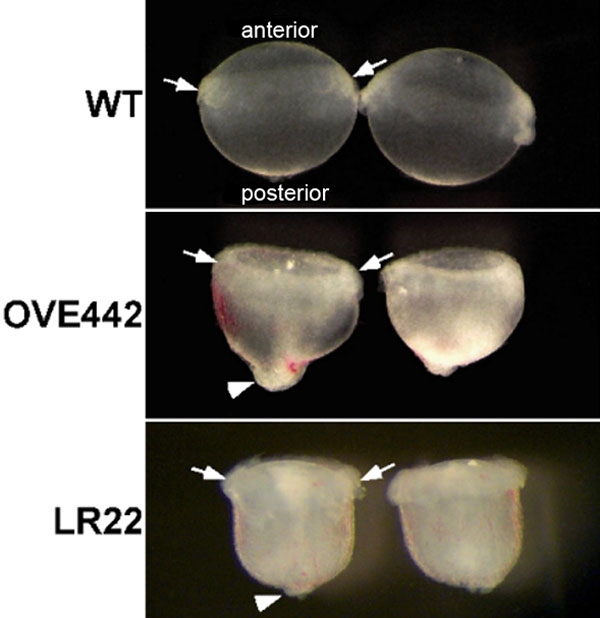
Lens morphology in wild type (WT), OVE442, and LR22 transgenic mice. Lenses were isolated from newborn (P0) mice for examination and photography. Some ciliary body tissues (arrows) were left on the lens to avoid damage or distortion to the lens during dissection. The WT lenses are transparent and spherical in shape. The transgenic lenses are cloudy and show a distorted shape. Additionally, there is an excess growth of the blood vessels at the posterior pole of the transgenic lenses (arrowheads).

The transgenic lenses from families OVE442 and LR22 were compared to those of wild type mice (Figure 2). Newborn (P0) wild type lenses were round and transparent, whereas the OVE442 and LR22 lenses were cloudy and did not have a normal spherical shape (Figure 2). Changes in lens shape were not apparent in the other transgenic families. In addition to the lens defects, transgenic mice in most of the families had abnormalities in their ocular vasculature. There was an overgrowth and persistence of the tunica vasculosa lentis (TVL), particularly around the posterior pole of the lens (Figure 2). The vascular defects in the transgenic mice will be described in another article.

**Figure 3:**
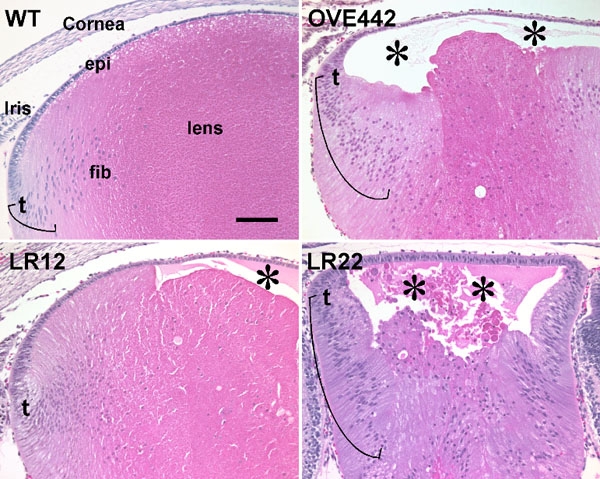
Lens histology in wild type (WT) and transgenic lines OVE442, LR12, and LR22 at birth. Fiber cell elongation was defective in all transgenic families, resulting in the presence of a subepithelial lumen (marked with asterisks). In the mildly affected line LR12, the lens polarity and the transitional zone (t) look normal. In the two severely affected lines (OVE442 and LR22), the transitional zone (t) is shifted anteriorly and is dramatically expanded (as shown in brackets).

Histological analysis showed that the severity of the lens defects correlated with the transgene expression level (Figure 3). Disruptions of normal lens structure were apparent at birth. In the mildly affected family LR12, the lens epithelium and cortex looked normal. However, morphologic changes were seen in the central fiber cells in the LR12 family and in the other transgenic families. Fiber cells failed to elongate completely and did not contact the anterior epithelial layer, resulting in the formation of a subcapsular lumen (Figure 3, asterisks). In addition, most of the central fiber cells retained their cell nuclei, indicating a disruption in normal fiber cell differentiation. Additional changes were observed in the severely affected families (OVE442 and LR22). First, the lens transitional zone was expanded (as marked with brackets in Figure 3). Second, there was an abnormal "bow pattern" (the arc-shaped path of fiber cell nuclei). Despite the severe defects in the lens fiber cells, the epithelial cells in transgenic mice looked relatively normal.

### Cell proliferation

**Figure 4:**
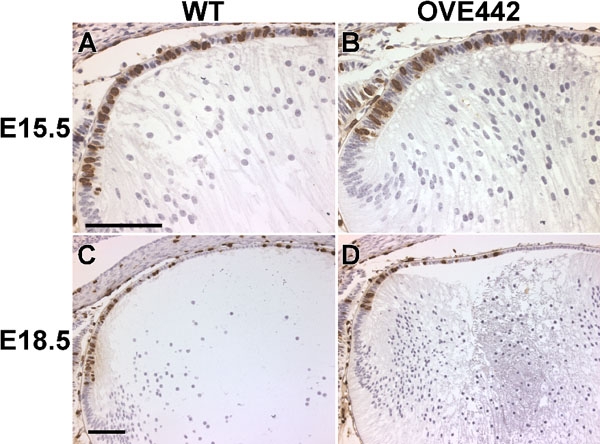
Cell proliferation in wild type (WT) and OVE442 transgenic mouse lenses. Lens cells at S-phase of the cell cycle were monitored with BrdU-labeling. BrdU-positive cells (brown nuclei) are distributed across the epithelial layer in the WT lenses (**A**, **C**) and OVE442 transgenic lenses (**B**, **D**). At E15.5 (**A**, **B**), the percentage of BrdU-positive nuclei is similar in the WT and transgenic lenses. At E18.5 (**C**, **D**), the percentage of BrdU-positive cells is slightly reduced in the transgenic lens, correlating with the phenotype of a reduced lens epithelial cell compartment.

The anterior shift of the lens transitional zone suggested that insulin overexpression might affect cell proliferation and differentiation in the transgenic lenses. BrdU labeling was performed to detect the lens cells in S-phase of the cell cycle (Figure 4). In wild type lenses, BrdU-positive cells were distributed across the lens epithelial layer (Figure 4A,C). At E15.5, the BrdU labeling pattern was essentially the same between the wild type and the transgenic lenses (Figure 4A,B and Table 1). At E18.5, when the lens phenotype began to develop, cell proliferation was affected (Table 1). The total number and percentage of BrdU-positive cells in the lens epithelial compartment were reduced in the OVE442 transgenic mice as compared to wild type mice (Table 1). This difference was statistically significant. In the wild type lens, BrdU-labeled cells were elevated near the germinative zone, where cell proliferation was most active (Figure 4C). Such an increase was not obvious in the transgenic lenses (Figure 4D). We also noted an anterior shift of the transitional zone in the transgenic lenses. This change was consistent with the reduction in the total number of lens epithelial cells. The data suggest that insulin overexpression results in an anterior shift of the differentiation zone in conjunction with a decrease in cell proliferation and a reduction of the lens epithelial layer.

**Table 1:** Comparison of BrdU-labeled cells in the wild type and transgenic mouse lenses.

Age	Genotype	Total number of lens epithelial cells	Total number of BrdU-labeled cells	Percentage of BrdU-labeled cells	Total number of sections examined
E15.5	Wild type	196±11	55±5	27.9±2.8	15
E15.5	OVE442	194±13	57±3	29.6±2.1	10
E18.5	Wild type	258±12*	60±13**	23.3±4.7***	10
E18.5	OVE442	210±10*	38±7*	18.1±3.9**	19

The single asterisk indicates a p<10^-10^; the double asterisk indicates a p<10^-6^, and the triple asterisk indicates a p<0.005. There was observed no statistical difference at E15.5 (all three p values were >0.1).

### Crystallin expression

**Figure 5:**
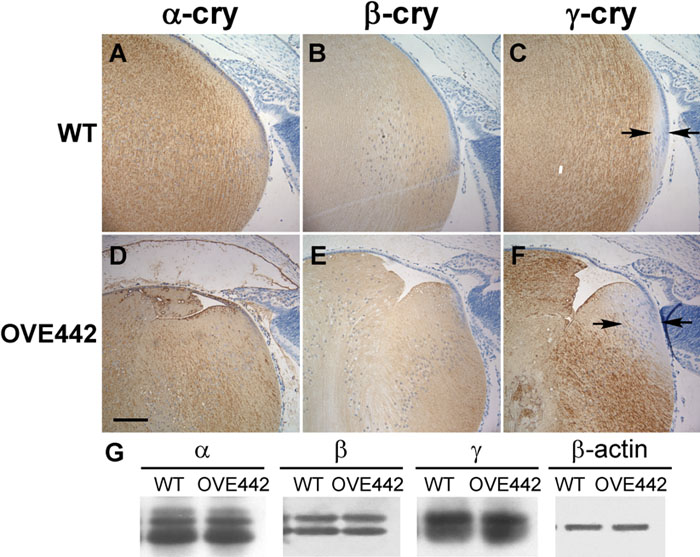
Crystallin expression detected by immunohistochemical staining and western blot. Eye sections of postnatal day 3 (P3) wild type (**A**-**C**) and OVE442 transgenic (**D**-**F**) mice were stained for α- (**A**, **D**), β- (**B**, **E**), and γ- (**C**, **F**) crystallins. In both genotypes, α-crystallin is expressed in all the lens cells, while β- and γ-crystallin are in the lens fiber cells. The space between the two arrows in (**C**) and (**F**) illustrate the area where γ-crystallin expression is absent. This area is expanded in the transgenic lens (**F**). With western blot analysis for α-, β-, and γ-crystallin (**G**), there was no detectable difference in the levels of crystallins between the wild type and transgenic mice. β-Actin was used as a control for protein loading.

To assess the differentiation state of the transgenic lens, crystallin expression patterns and levels were examined in wild type and OVE442 transgenic mice (Figure 5). We found that α-crystallin expression was not altered (Figure 5A,D). In the transgenic lenses, β-crystallin expression was activated slightly earlier than normal (Figure 5E). This finding is consistent with the observation that the transitional zone was shifted anteriorly in the transgenic lenses. In contrast to β-crystallin, γ-crystallin expression was delayed in the transgenic lenses (Figure 5F), suggesting that more fiber cells were retained in an initial differentiation state, a pattern consistent with the expansion of the transitional zone in the transgenic lenses (Figure 3). Although the transgenic lenses showed altered expression patterns for β- and γ-crystallin, the total protein levels for all three types of crystallins were similar in the wild type and transgenic lenses (Figure 5G).

### Fiber cell differentiation and maturation

**Figure 6:**
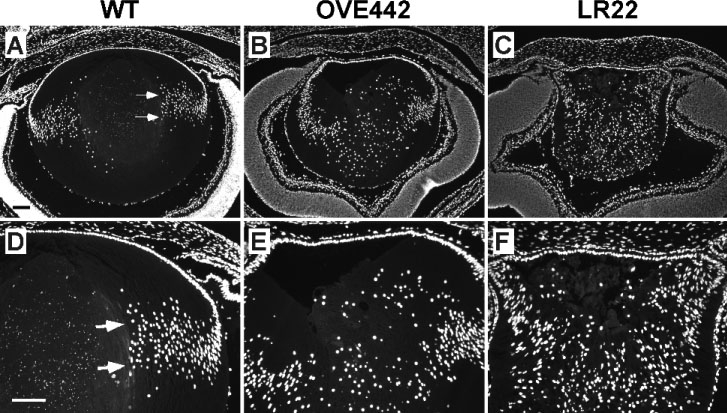
Fiber cell denucleation in newborn wild type (WT), OVE442 and LR22 mouse lenses. Newborn eye sections were stained with DAPI to reveal the distribution of cell nuclei. In the WT lens (**A**, **D**), fiber cell maturation is associated with denucleation and formation of a nuclear-free zone (arrows mark the margin of the area). In OVE442 (**B**, **E**) and LR22 (**C**, **F**) transgenic lenses, fiber cell nuclei are present across the entire lens section.

During later stages of differentiation, fiber cells lose their nuclei (denucleate) and intracellular organelles to form the organelle-free zone (OFZ) at the lens core [4]. Histology data indicated impairment of denucleation in the transgenic lenses (Figure 3). To confirm this observation, lens sections of newborn wild type, OVE442, and LR22 mice were stained with the nuclear dye DAPI (Figure 6). The OFZ was visible in the wild type lenses (Figure 6A,D). In contrast, cell nuclei in the transgenic lenses were distributed across the entire lens (Figure 6B,C,E,F), suggesting that insulin expression inhibits fiber denucleation.

**Figure 7:**
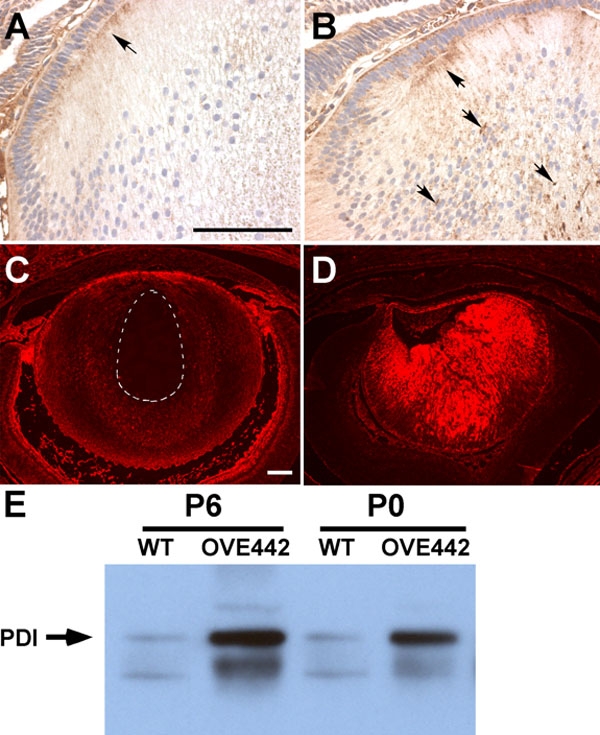
Detection of COX and PDI proteins. In the WT E18.5 lens (**A**), COX protein was detected in the epithelial cells and in the secondary fiber cells at the cortex. Strong COX-immunostaining was seen in the anterior tips of the secondary fiber cells (**A**, arrow). COX-staining was enhanced in the transgenic lens (**B**), particularly in the central region of the lens, where concentrated COX-staining was seen (**B**, downward arrows). PDI (rhodamine fluorescence in red) is highly expressed in the lens epithelial cells and cortical fiber cells in WT lens (**C**). PDI staining is lost in the central fiber cells (**C**, circled area). In contrast, PDI expression persists in all the lens fiber cells in OVE442 transgenic mice (**D**). **E**: PDI western blot analysis on newborn (P0) and postnatal day 6 (P6) lens. Compared to the PDI protein level in the WT lenses, expression is increased significantly in the OVE442 transgenic lenses (**E**, arrow). The lower band is from the reactivity of the secondary antibody to mouse endogenous immunoglobulin protein.

To examine whether intracellular organelle degradation was also affected in the transgenic lenses, sections were stained for cytochrome c oxidase (COX) subunit I and protein disulfide isomerase (PDI), which are markers for mitochondria and endoplasmic reticulum (ER), respectively [44,45]. The mitochondrial protein COX was present in both lens epithelial cells and cortical fiber cells, with high-intensity staining at the anterior margin of the fiber cells (Figure 7A). COX staining was reduced in mature fiber cells. Compared to the wild type lens, the transgenic lenses exhibited enhanced COX staining (Figure 7B), particularly in the central region, where strong COX staining was detected (downward arrows in Figure 7B). The ER protein PDI was expressed in wild type epithelial cells and cortical fiber cells. Fiber cells at the center were PDI-negative in the wild type lens (Figure 7C). In the transgenic lenses, PDI was expressed in both epithelial cells and fiber cells, and the level was increased, particularly in the fiber cells (Figure 7D). Western blot analysis confirmed this observation (Figure 7E). Taken together, our data suggest that elevated insulin signaling in the lens interferes with fiber differentiation and maturation.

### Signaling pathways affected by insulin overexpression

**Figure 8:**
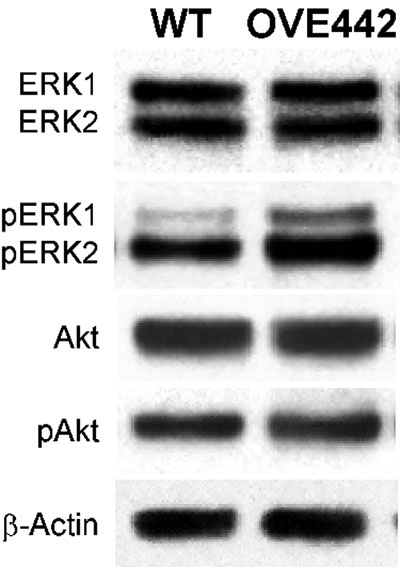
ERK and Akt protein and phosphorylation levels. Water-soluble proteins from newborn lenses were used for western blotting to detect ERK, phosphorylated ERK (pERK), Akt, and phosphorylated Akt (pAkt). The pERK levels (both pERK1 and pERK2) are increased 2-3 fold in the transgenic lenses while the ERK protein levels remain the same as that in the WT lenses. No change in Akt or pAkt levels were detected. The analysis was repeated on three independent samples and the results were consistent. β-Actin was used as a control for protein loading.

Insulin exerts its biological activity through activation of the insulin receptor (IR). In many different cell types, IR activation turns on downstream signaling pathways involving Ras and PI3K, which subsequently activate the Akt kinase and/or the Raf-MEK-ERK cascade. To explore insulin-activated signaling in the mouse lens, we analyzed the phosphorylation levels of ERK and Akt by western blot analysis (Figure 8). The levels of phosphorylated (active) ERK (both pERK1 and pERK2) were increased 2-3 fold in the transgenic lenses while the total ERK protein level was unchanged. Surprisingly, phosphorylated and total Akt protein levels in the transgenic lenses were the same as those in the wild type lenses. These results suggest that the phenotypic changes in the insulin transgenic lenses are mediated through the ERK, rather than the Akt, signaling pathway.

**Figure 9:**
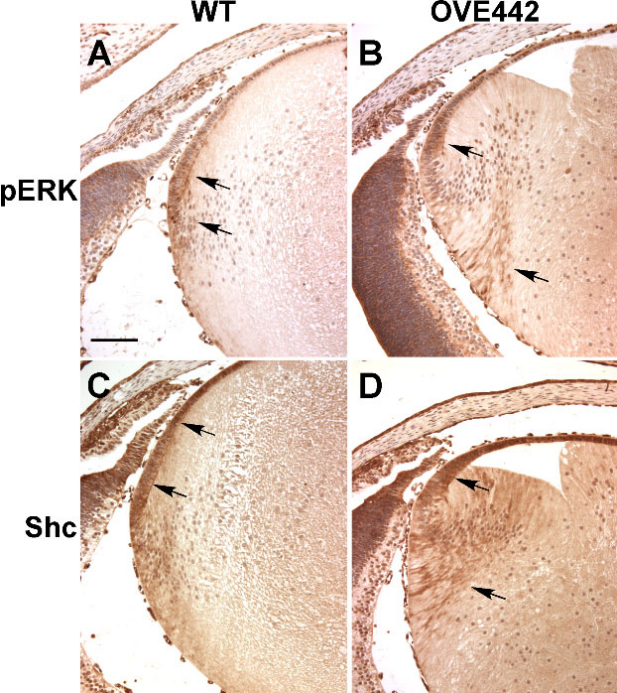
Immunohistochemical staining for pERK and Shc. In the WT E18.5 lens, pERK immunoreactivity was detected in the lens epithelial cells at the germinative zone and in the newly differentiating fiber cells (**A**, arrows). In the OVE442 transgenic lens, the pERK-staining area is expanded in the fiber cells (**B**, arrows). The immunostaining pattern for the adaptor protein Shc is also changed in the transgenic lens. In the WT lens, Shc is most strongly expressed in the lens epithelial cells (**C**, arrows). In the OVE442 transgenic lens, strong Shc staining was detected not only in the epithelial layer but also in the cortical fiber cells (**D**, arrows).

Increased phospho-ERK levels in the transgenic lenses were further examined by immunohistochemical staining (Figure 9A,B). In the E18.5 wild type lens, pERK immunoreactivity was detected both in the cytoplasmic and nuclear compartments of cells at the germinative and transitional zones (Figure 9A). Strong pERK staining was also found at the epithelial-fiber cell apical junctions. The pERK staining pattern was altered in the transgenic lenses, with the lens cortex showing more pERK-positive fiber cells (Figure 9B). This change likely accounts for the increased pERK level in the western blot (Figure 8).

Shc and IRS-1 are two receptor adaptor proteins. These adaptor proteins bind to the activated receptors at tyrosine docking sites, are phosphorylated and, in turn, recruit SH2-domain-containing signaling molecules such as Grb2 and the p85 regulatory subunit of PI3K [46,47]. Immunostaining for Shc detected protein expression in the epithelial cells and in young fiber cells at the cortical region in the wild type lens (Figure 9C). The Shc protein level sharply decreased to the background level as fiber cell differentiation proceeded. In the transgenic lens, the Shc-positive region was expanded into the cortical fiber cells (Figure 9D). In contrast to Shc, IRS-1 protein was expressed at a much higher level in lens fiber cells than in epithelial cells, and the staining pattern was similar in wild type and transgenic lenses (data not shown).

## Discussion

Lens epithelial cells in explants can respond to insulin and IGF stimulation [9,12,48], suggesting that growth factors in the insulin/IGF family could potentially regulate cell proliferation and differentiation during normal lens development. Transgenic mice that overexpress IGF-1 in the lens have been previously generated [14]. IGF-1 by itself did not function as a differentiation factor, but it did modify the architecture of the lens by causing the germinative and transitional zones to extend toward the posterior pole of the lens. In the current study, we have generated transgenic mice that overexpress insulin in lens fiber cells and in both lens epithelial cells and fiber cells [38]. We found that insulin and IGF-1 transgenic mice have similar yet distinct phenotypes. Neither insulin nor IGF-1 was sufficient to induce premature differentiation of the lens epithelial cells in vivo. This result was consistent with the findings in rat lens explants where neither IGF nor insulin alone induced epithelial-to-fiber differentiation [11-13]. Elevated levels of insulin or IGF-1 produced slight modifications of the cell proliferation pattern, but the lens epithelium in both genotypes remained as a monolayer. This result is different from the effect of PDGF-A on lens epithelial cells [8,49,50].

In both the insulin and IGF-1 transgenic mice, fiber cell maturation was perturbed and the transitional zone was expanded. An interesting difference was the location of the lens germinative and transitional zones. In the IGF-1 mice, the lens epithelial compartment was expanded and the germinative zone was localized more posterior than in the wild type lens [14]. In contrast, the lens transitional zone was moved anteriorly in the insulin transgenic mice. These changes correlated with slight changes in BrdU-labeling in the IGF-1 and insulin mice. Our data suggest that IGF and insulin are not direct inducers of fiber cell differentiation. Instead, members of the insulin growth factor family may play supportive roles in fine tuning lens growth and differentiation to achieve a normal lens structure and shape.

It has been shown that one role of Shc in cells is to promote the formation of a Shc-Grb2-SOS complex, which can activate the Ras-Raf-MEK-ERK kinase cascade [46]. Previously, we demonstrated that cell proliferation during lens development depends on the activity of the Ras-ERK signaling pathway [51]. Thus, the slightly enhanced cell proliferation signal in IGF-1-overexpressing transgenic mice could be explained by an increase in Ras-ERK signaling activity. In the lenses of the insulin transgenic mice, we showed that ERK activity and Shc expression were enhanced in the differentiating fiber cells (Figure 8 and Figure 9). In contrast, phosphorylated Akt and IRS-1 expression were not altered (Figure 8, and data not shown). It is possible that IR activates additional signaling pathways, which override the Shc-ERK proliferation signal and initiate an early epithelial-to-fiber transition. Analyzing additional signaling pathways may help resolve this paradox.

In most of the transgenic families (except LR20), fiber cell differentiation was disrupted by insulin overexpression. Fiber elongation was incomplete and cell nuclei, mitochondria, and ER were retained in the central fiber cells. Our data suggest that insulin signaling may antagonize the endogenous signals that are required for late-stage fiber differentiation. At present, the molecular mechanisms that regulate fiber cell terminal differentiation, such as denucleation and organelle degradation, have not been defined. Apoptosis-related proteins may have a role in this process [52,53]. It was recently suggested that the ubiquitin-proteasome pathway might be involved in the breakdown of intracellular organelles during fiber differentiation [54]. We have not investigated whether these activities are affected in the transgenic lenses. Based on studies in other systems, IGF and insulin are known to be inhibitors of cell death. Our western blot analysis and immunostaining showed that ERK phosphorylation was increased, whereas the pAkt level was unaffected in the insulin transgenic lenses. Therefore, the increased ERK activity could be contributing to the disruption of the fiber differentiation program.

In summary, we have demonstrated that insulin overexpression in the lens is not sufficient to initiate epithelial-to-fiber cell differentiation, but it can alter lens growth and fiber cell maturation. Combined with the previous study of the IGF-1 transgenic mice, our data suggest that insulin and IGF-1 could have overlapping yet distinct functions in lens development. We propose that, during lens development, neither IGF-1 nor insulin is a differentiation inducer. Instead, they function as modifiers of fiber cell morphology. The balance between IGF-1 and insulin signaling in the lens may help to finely tune lens growth and maturation. Additionally, our study suggests that fiber cell terminal differentiation requires downregulation of insulin-activated signaling pathways. Elevated ERK activity may antagonize the endogenous apoptotic-like or proteolytic activities that are involved in late stage fiber cell differentiation.
